# A qualitative study on the experiences of caregivers on menstrual management of adolescent girls with developmental disabilities in selected special schools of Karveer Taluka, Maharashtra

**DOI:** 10.1186/s12905-025-04029-y

**Published:** 2025-10-06

**Authors:** Madhura Meghraj Bhosale, Anusree Prabhakaran, Arathi P Rao

**Affiliations:** Department of Global Public Health Policy and Governance, Prasanna School of Public Health, Manipal Academy of Higher Education, Manipal, India

**Keywords:** Developmental disabilities, Adolescent girls, Menstruation, Caregivers, Qualitative

## Abstract

**Background:**

Adolescent Girls with Developmental Disabilities (AGDD) face unique challenges in menstruation management, requiring care and support from both informal and formal caregivers. Limited research has explored the experiences of these caregivers in managing AGDD’s menstrual health, particularly in this study setting.

**Objective:**

This study seeks to explore the experiences and perspectives of the informal and formal caregivers on the menstruation management of adolescent girls with developmental disabilities in selected special schools in Karveer Taluka, Maharashtra.

**Methods:**

A qualitative study was conducted among informal caregivers (family members) and formal caregivers (special school staff) of those AGDD, who were aged 10–19 years, had attained menarche, and were attending three selected special schools of Karveer Taluka, Maharashtra, India. In-depth interviews (IDI), in the local language, Marathi, using an IDI guide developed based on a conceptual framework, were conducted among 14 informal and 13 formal caregivers. Interviews were audio-recorded with consent and transcribed verbatim. A thematic analysis using deductive and inductive approaches was carried out using MAXQDA software. Some codes and themes were deductively generated from the interview guide/conceptual framework, and others were generated inductively from the data. The transcripts were coded line by line and later grouped under relevant categories. Categories were grouped into subthemes, and broader themes were derived from the subthemes. The themes were interpreted and analyzed, and appropriate conclusions were drawn.

**Results:**

Four deductive and one inductive theme were generated: The deductive themes are challenges faced by informal caregivers in managing menstruation of AGDD, patterns of menstrual acceptance and behavioural changes among AGDD, long-term concerns of the informal caregivers and perspectives on menstrual management, and recommendations given by the informal and formal caregivers for supporting AGDD. The inductive theme generated is the role of formal caregivers in training AGDD in menstruation management.

**Conclusion:**

The study underscores the critical role of informal and formal caregivers and their challenges in managing AGDD’s menstrual health, highlighting the need for targeted training and support programs.

**Supplementary Information:**

The online version contains supplementary material available at 10.1186/s12905-025-04029-y.

## Background

“Developmental Disabilities (DD) are conditions characterized by an impairment in physical, learning, language, or behaviour areas”, including Intellectual Disability, Language Disorder, Learning Disorder, Fragile X Syndrome, Attention-Deficit/Hyperactivity Disorder (ADHD), Cerebral Palsy, Down Syndrome, and autism spectrum disorders [1]. Globally, an estimated 317 million children and adolescents suffer from health conditions that lead to DD, and the prevalence varies from 7.5% among those who are below five years and 13.9% among those who are between 15 and 19 [2]. Low-and middle-income countries bear a higher burden [3]. Approximately 0.93% of the population of India is affected by DD [4].

As individuals with DD enter adolescence, a crucial phase for physical changes and cognitive and psychosocial development, the challenges related to their disabilities become more pronounced [5]. Adolescent Girls with Developmental Disabilities (AGDD) face unique challenges as they navigate through puberty and the onset of menstruation [6, 7]. Many AGDD lack the capacity to understand menstruation-related issues. Hence, they require ongoing assistance from family members or other caregivers. Menstruation in AGDD raises challenges for the informal caregivers who take care of the individuals in the home and formal caregivers who are involved in the day-to-day activities of the individuals in other settings, such as classrooms or schools [8].

Most existing studies have been conducted within community settings, focusing primarily on informal caregivers like parents [9–11]. However, the experiences of formal caregivers in specialized settings, where structured routines and educational interventions are integral to the girls’ development, remain largely unexplored. Special schools serve as key institutions where they not only support the academic needs of developmentally disabled adolescents but also play a critical role in managing physical changes such as menstruation. To our knowledge, minimal research has been conducted in this particular setting. Hence, the objectives of this study are.

1. To explore the experiences of the informal caregivers in managing the menstrual health of adolescent girls with developmental disabilities who are students in the selected special schools of Karveer taluka, Maharashtra.
2. To explore the perceptions of formal caregivers of adolescent girls with developmental disabilities regarding their menstrual health in selected special schools of Karveer taluka, Maharashtra.

This study is of utmost importance as it aims to bridge the knowledge gap by exploring the experiences of both informal caregivers (parents/guardians) and formal caregivers (special school staff) in managing the menstrual health of AGDD. By exploring their perspectives, experiences, concerns, strategies, and challenges, the findings can provide valuable insights into improving support systems and developing targeted interventions to better equip both groups of caregivers in promoting menstrual health and well-being for these girls.

## Methods

This section outlines how the study was carried out, including details about the methodological outline, research team, study sample, methods of approach, sample size, study setting, data collection, and data analysis.

### Methodological outline

The study followed a descriptive qualitative design [12], underpinned by interpretivism [13]. Data was collected through in-depth interviews using a tool developed based on a conceptual framework [14] and some additional questions. Analysis involved deductive (guided by the framework/IDI guide) and inductive (emerging from the data) approaches.

### Research team

The research team comprised three female members: MMB, APR, and AP. MMB is a public health researcher, APR is a gynaecologist with a doctorate in public health, and AP is a doctoral researcher. All three members are trained in qualitative research methodologies.

### Study sample

This study included informal and formal caregivers of AGDD, who were aged 10–19 years, had attained menarche, and were attending three selected special schools of Karveer Taluka, Maharashtra. Informal caregivers referred to parents or guardians who were primarily responsible for caring for the girls at home. Formal caregivers included special school teachers and caretakers. Special school teachers were engaged with the girls throughout the day in the school setting, communicated with them, educated the girls, provided skill development training, observed their behaviour changes, communicated with parents, etc. They are responsible for monitoring, supporting, and coordinating care for the AGDD. Though they are not always involved in providing direct physical care, such as toileting and menstrual management, they are well-positioned enough to reflect on how menstruation was managed in the school environment and the support needs of AGDD during this time, and are hence considered for the study. Caretakers included those formal caregivers who provided direct physical care, such as helping AGDD change pads, feeding, helping with toileting, etc. Because of their involvement in the intimate care of AGDD during menstruation, they were included in this study. Caregivers who were both working as special school staff in any of these three schools and taking care of their own developmentally disabled daughter (those with the role of formal and informal caregiver) were not included in the study.

### Method of approach

After obtaining administrative permission from the chairperson of the schools, MMB approached the informal caregivers over the phone and the formal caregivers in person, explained the purpose of the research, researchers ' interest in the topic, and invited them to participate in the study. Prior to data collection, rapport was built with the participants.

### Sample size

A purposive sampling method was used to recruit participants. A total of 20 informal and 18 formal caregivers were approached; however, only 14 informal and 13 formal caregivers participated in the study. Five informal caregivers cited lack of time as the reason for non-participation; one was unwilling due to the overwhelming nature of her caregiving responsibilities. Additionally, five formal caregivers were unavailable in the school at the time of data collection and were not included in the study.

### Study setting

This study was conducted in three special schools of Karveer Taluka, Kolhapur district of Maharashtra, India. These three special schools catered to those aged 5–19 years, with moderate to severe developmental disabilities, including intellectual disability, cerebral palsy, autism spectrum disorders, and Down syndrome. These institutions were selected through a snowball sampling method. We approached one of the special schools in Kaveer Taluk, Maharashtra, which agreed to participate. The senior officer in this special school referred us to two other special schools with a similar population profile, which also consented to be part of the study. The data from informal caregivers were collected from the school or at their residence, based on convenience. Data from all formal caregivers were collected from the school premises.

### Data collection

Participant information sheets were provided, and informed consent was obtained. An IDI guide was used to collect data. The IDI guide was developed by referring to the conceptual framework “caregiving process and caregiver burden among pediatric population”, proposed by Raina et al., 2004. [14] It guided the development of the IDI guide by helping to identify areas such as caregiving needs, the burden of managing menstruation of AGDD, and the support systems available. However, we also included additional questions outside the framework to capture broader aspects of caregivers’ experiences that we felt were relevant but not covered in the conceptual framework. Once developed, the IDI guide was content validated by two subject experts to ensure the questions were appropriate and clear. After content validation, pilot interviews among one informal and one formal caregiver were conducted. Based on the responses from the pilot interviews and after consultation with the subject experts, the IDI guide was refined. The interview guide developed is attached as supplementary file 1. All the interviews were conducted in the local language, Marathi, by MMB. No one else was present besides the participant and MMB. Duration of interviews ranged between 20 and 40 min. Field notes were made during the interview. Interviews were audio recorded after obtaining prior consent from the participants and later transcribed and translated into English. Interviews were conducted until data saturation was obtained.

### Data analysis

Data saturation was observed after conducting 11 interviews among informal caregivers and 10 interviews among formal caregivers. However, 14 and 13 interviews among informal and formal caregivers, respectively, were conducted to ensure all the vital information was captured. No repeat interviews were carried out.

The audio recordings were transcribed verbatim and translated. The transcripts were saved in a password-protected computer. It was not returned to participants. Later, the transcripts were imported into the MAXQDA 2022 software for further qualitative analysis. A thematic analysis, using both deductive and inductive approaches, was conducted. The transcripts were coded line-by-line by MMB. Deductive codes were developed based on the conceptual framework and the additional questions in the interview guide (which were not based on the conceptual framework). Inductive codes were generated directly from the data, allowing us to capture the participants’ narratives that were not anticipated in the interview guide. To ensure consistency and validity, APR and AP further reviewed the transcripts and codes. The codes were later grouped under relevant categories, and sub-themes and broad themes were generated. The themes were interpreted and analysed, and appropriate conclusions were drawn.

To maintain the anonymity of the research participants, each participant was assigned a unique identification number. To maintain the confidentiality of the data, access to the recordings and transcripts was provided to the research team only. Participants were not involved in feedback.

## Results

Table 1 contains the details of the informal caregivers and Table 2 contains the details of the formal caregivers.

**Table 1:** Characteristics of informal caregivers

Informal caregiver ID	Sex	Age (years)	Occupation	Education	Area of living	No. of family members	Age of AGDD at the time of interview	Relationto the AGDD	AGDDs’ Disability type	Intellectual impairment percentage (%)	Year in which menarche was attained	Menstrual Products Used
P1	F	35	Housewife	12th grade	Rural	5	18yrs	Mother	Autism	90%	2016	Cloth
P2	F	40	Tailor	12th grade	Urban	4	14yrs	Mother	Cerebral Palsy	75%	2022	Cloth
P3	F	40	Housewife	Post-graduate	Urban	4	16yrs	Mother	Intellectual disability	52%	2021	Sanitary napkin
P4	F	41	AnganawadiWorker*	12th grade	Urban	4	18yrs	Mother	Cerebral palsy	66%	2018	Sanitary napkin
P5	F	40	Housewife	Post-graduate	Rural	7	13 yrs	Mother	Cerebral palsy	50%	2022	Diaper
P6	F	36	Housewife	7th grade	Urban	5	17 yrs	Mother	Down Syndrome	52%	2016	Sanitary napkin
P7	F	38	Housewife	10th grade	Urban	6	13 yrs	Mother	Cerebral palsy		2021	Sanitary napkin
P8	F	34	Housewife	10th grade	Urban	5	17yrs	Mother	Autism	60.17%	2017	Sanitary napkin
P9	F	36	Housewife	Post-graduate	Urban	4	15yrs	Mother	Intellectual disability	67%	2019	Sanitary napkin
P10	F	47	Working	Graduate	Urban	3	18 yrs	Mother	Cerebral Palsy	60%	2019	Sanitary napkin
P11	F	54	Housewife	8th grade	Urban	3	14yrs	Mother	Autism	65%	2021	Period panty
P12	F	41	Housewife	Graduate	Urban	4	17yrs	Mother	Intellectual disability	90%	2018	Sanitary napkin
P13	F	39	Housewife	12th grade	Rural	5	16yrs	Mother	Intellectual disability	58%	2021	Cloth
P14	F	35	Maid	10th grade	Urban	4	17 yrs	Mother	Autism	75%	2018	Pad

Anganwadi worker: A frontline community health worker employed in Anganwadi as part of the Integrated Child Development Scheme (ICDS) in India.

*Anganwadi:A Child care centre in India that provides a range of services for young children (3-6 years) and mothers, including health checkups, nutrition, and pre-school education

**Table 2:** Characteristics of formal caregivers

Formal Caregiver ID	Sex	Age (years)	Occupation	Area of living	No of family members	School ID
P1	F	53	Special teacher	Urban	3	School A
P2	F	56	Special teacher	Urban	4	School A
P3	F	49	Special teacher	Rural	6	School A
P4	F	53	Special teacher	Rural	4	School A
P5	F	44	Special teacher	Urban	4	School A
P6	F	31	Special teacher	Urban	3	School B
P7	F	33	Special teacher	Urban	4	School B
P8	F	45	Special teacher	Urban	5	School B
P9	F	38	Caretaker*	Urban	4	School B
P10	F	38	Special teacher	Urban	4	School B
P11	F	42	Special teacher	Urban	4	School C
P12	F	34	Special teacher	Urban	3	School C
P13	F	45	Special teacher	Urban	3	School C

*Caretaker:In the context of special schools, a caretaker refers to non-teaching support staff, called ayas or maids, who provide physical care to AGDD, such as feeding, helping them in managing personal hygiene, menstruation management, such as changing pads, etc

Five themes were generated from this study.

1. Challenges faced by informal caregivers in managing the menstruation of AGDD
2. Patterns of menstrual acceptance and behavioural changes among AGDD
3. Role of formal caregivers in training AGDD in menstruation management
4. Long-term concerns of informal caregivers and perspectives on menstrual management
5. Recommendations given by informal and formal caregivers for supporting AGDD

Table 3 shows a summary of each theme and its key findings.

**Table 3:** Themes and key points

Themes	Key points
Theme 1: challenges faced by informal caregivers in managing the menstruation of AGDD	• AGDD’s inability to manage menstruation independently • AGDD’s resistance towards menstrual products • Need for constant supervision by informal caregivers • Embarrassment from relatives and society
Theme 2: patterns of menstrual acceptance and behavioural changes among AGDD	• Behaviour changes observed during menstruation: irritability, discomfort, being calm and silent • Behaviour changes associated with puberty: Increased interest in personal appearance, increased attraction towards the opposite sex
Theme 3: role of formal caregivers in training AGDD in menstruation management	• Reduce parental support to promote independence in AGDD • Treat AGDD the same as their typically developing peers • Keeps extra dresses and sanitary pads in school for AGDD • Peer-learning: Caregivers verbally instructed AGDD who had already begun menstruating on how to manage their menstruation, and then encouraged these girls to demonstrate the process to others who had not yet started menstruating. • Tissue paper method: Demonstrating the AGDD to use a tissue paper and put a red mark if they notice they are menstruating – helpful for those who have difficulty in communication
Theme 4: long-term concerns of informal caregivers and perspectives on menstrual management	• Informal caregivers expressed concerns about the future of their child in their absence • One has consulted doctors to seek the possibility of removing the uterus of their daughter due to the fear of future sexual abuse • The majority opposed surgical options such as hysterectomy
Theme 5: recommendations given by informal and formal caregivers for supporting AGDD	• Educating and training AGDD • Communication and interaction among informal and formal caregivers • Provision of quality menstrual products • Infrastructure and institutional support • Policy and financial protection

### Theme 1: challenges faced by informal caregivers in managing the menstruation of AGDD

The AGDD in this study had a range of disabilities such as intellectual disability (*n* = 4), autism spectrum disorder (*n* = 3), cerebral palsy (*n* = 5), and Down syndrome (*n* = 1), and their intellectual impairment ranged from 52 to 90%, and all of them were dependent, either partially or completely, on their caregivers for managing menstruation; none were able to manage it independently. Informal caregivers provided physical and emotional support to the girls and trained them in handling menstruation. Due to the demanding caregiving needs, informal caregivers of AGDD experienced both physical and emotional challenges during handling the menstruation of their child. They expressed concerns related to the girls’ inability to understand and handle menstruation independently, difficulty due to the additional responsibility, and, embarrassment from the family and society.

*“I was afraid because what if her dress gets stained. She won’t understand it.”* –(Participant 7, Informal Caregiver)

*“She cannot understand anything right? I used to do everything for her already and now these became an extra responsibility”* - (Participant 5, Informal Caregiver)

Informal caregivers expressed that the demands and challenges of managing menstruation negatively impacted their freedom and quality of life. Due to girls’ resistance to using menstrual pads or other menstrual products, caregivers have to provide extra care and support to the girls, which places an additional caregiving burden on informal caregivers.

*“At home*,* I used to change 10–15 pairs of clothes for her. Because she didn’t want to wear a whisper”* (Participant 2, Informal Caregiver).

Few informal caregivers reported that menstruation demanded constant supervision by the informal caregivers at home.

*“We can’t go out when she gets her period. I did not go anywhere for 15 days”* (Participant 3, Informal Caregiver).

*“If any guest comes and she is on her period… Someone at home must keep an eye on her and care for her.”* (Participant 1, Informal Caregiver)

The demanding caregiving responsibilities of managing menstruation, coupled with the embarrassment from relatives and society, resulted in emotional strain in informal caregivers. Few informal caregivers shared that the responses from family members and close relatives regarding the menarche of their child were disturbing.

*“ahhhh relatives did a lot of hammering… that your responsibility has increased*,* what if someone made her pregnant? Give her some injection and kill her.”* (Participant 4, Informal Caregiver)

In contrast, another informal caregiver experienced curious questioning about the menstruation of her daughter from society,

*“People constantly asked me why isn’t your daughter having her periods yet.”* (Participant 14, Informal Caregiver)

Few informal caregivers believed that menarche happened at a relatively early age and they wished that these girls would have experienced menarche later.

*“I was surprised that this happened too early. I had a wish that at least these girls shouldn’t get it too soon.” (Participant 2*,* Informal Caregiver)*.

Many informal caregivers reported that the support from male parents and siblings of AGDD was crucial as they navigated through the challenges. Without any hesitancy, fathers, brothers, and sisters took on responsibilities that included changing pads, properly discarding them, and providing guidance or reminders about menstrual hygiene.

*“Her father puts pampers on her. Even I get my own periods*,* so at that time*,* who helps me? That is her father who does it. "* (Participant 3, Informal Caregiver).

*“And even her sister supports her; she tells her that” “Didi you got your periods? Mummy told you right to go and change your pads. Now go take that pad and do it.” Even she instructs her at times”* (Participant 8, Informal Caregiver).

*“Yes he (the girl’s brother) changes my daughter’s pads then he washes that pad and throws it in the dustbin after rolling it in a wrapper.”* (Participant 4, Informal Caregiver).

This theme points out that informal caregivers reported both emotional and physical challenges associated with managing the menstruation of AGDD. Most girls required full support from the informal caregivers, which exacerbated the burden on caregivers. Caregivers reported increased burden due to the girls’ lack of understanding about menstruation, resistance to menstrual products, need for constant supervision, embarrassment, and judgment from relatives and society. Despite these, support from family members, especially both male and female siblings and fathers of the AGDD, was a relief to a few informal caregivers.

### Theme 2: patterns of menstrual acceptance and behavioural changes among AGDD

Both informal and formal caregivers noted that AGDD often gets irritated during menstruation. However, some girls become hyperactive, and some become calm during menstruation.

*“She is very much irritable before her periods*,* very much as in beating*,* hitting*,* or being restless all the time”* (Participant 1, Informal Caregiver)

*" If the clothes of those girls get wet*,* then it bothers them*,* then they get irritated. Some girls don’t want it and want it to be removed.”* (Participant 8, Formal caregiver).

In contrast to their usual energetic behaviour, some became notably quiet and less physically active during menstruation.

*“… the rest of the time*,* she is always hyper*,* but at that time*,* she remains quiet.”* (Participant 7, Informal Caregiver).

In addition to the mood changes, AGDD also experiences the same desires for attractiveness and self-expression as typically developing peers. Caregivers noted an increased interest in personal appearance and grooming among AGDD.

*“….Now I have noticed it in this year. She applies powder*,* wears different earrings*,* wears necklaces.”* (Participant 8, Informal Caregiver).

On the other hand, formal caregivers noted that adolescents begin to understand physical touch and may experience attraction during this stage. They observed behavioural changes, such as increased attention towards peers of the opposite sex and mischievous interactions with them.

*“…very much attraction has increased. Be it anyone even a boy or if she likes any person*,* she gets attracted to them”* (Participant 9, Informal Caregiver)

*“…instead of being with girls they start chit chatting with boys or touching them” (Participant 13*, Formal caregiver*)*

*“They try to hug male staff*,* then we have to tell them he is our sir*,* so you should not hug him like that.”* (Participant 7, Formal caregiver)

This theme highlights that both informal and formal caregivers noted that AGDD expressed different behavioural changes during menstruation, including irritability, discomfort, and in some cases, calmness. In addition to these, they also observed that AGDD became more interested in personal appearance and attraction towards the opposite sex. This suggests that AGDD also goes through the same physiological and social changes just as their typically developing peers.

### Theme 3: pole of formal caregivers in training AGDD in menstruation management

Formal caregivers, such as special school teachers and caretakers, played a significant role in training the girls to manage menstruation. They emphasized the importance of promoting the independence in these girls to manage menstruation. Similarly, they have explained the different methods they employed to train the girls on menstruation management.

One teacher emphasized the importance of reducing parental support during menstruation care, encouraging parents to train their daughters to manage their periods independently.

*“Parents also help them at home; their support is maximum. We talk to the parents to reduce that support and train them.”* (Participant 1, Formal caregiver).

In line with this, another formal caregiver also mentioned the importance of treating the AGDD as similar to their typically developing peers when it comes to menstruation.

*“Do your other girls stay at home during their periods? No*,* right. then why do you keep this girl at your home?”* (Participant 5, Formal caregiver).

Additionally, schools took steps to ensure that AGDD had the necessary resources for menstrual care during school hours. Many formal caregivers mentioned keeping extra dresses and pads readily available for the girls.

*“We keep extra dresses and pads in school. So if we check and see that it is necessary now to change her pad we ask our caretakers to change her pads”* (Participant 13, formal caregiver).

In the special school setting, formal caregivers employed different tailored approaches based on the intellectual impairment percentage of the AGDD to teach them about menstrual hygiene and management. It included verbally instructing, demonstrating, peer learning, and using visual aids such as tissue papers. One of the special school teachers mentioned that these girls are fond of learning through imitation. They used an approach where the teachers verbally instructed the AGDD who had already experienced menstruation, and these girls demonstrated accordingly.

“*… we include two girls who menstruate and two girls who don’t. Now for the girls who menstruate*,* we verbally instruct them*,* and then these girls show accordingly.”* (Participant 4, Formal caregiver).

The other teacher used a strategy for instructing girls to notify their family members or teachers by using red marks to replicate period stains on tissue paper.

*“…. we put a red mark on tissue paper and tell them if you see this kind of mark on your dress*,* you should go immediately and inform your mother*,* sister*,* grandmother*,* auntie and in school*,* me or caretaker.”* (Participant 11, Formal caregiver).

This theme highlights that formal caregivers in a special school setting play a crucial role in training AGDD in managing their menstruation. They focus on promoting independence, reducing parental support, and put efforts in convincing parents to treat AGDD and same as their typically developing peers. They ensured that AGDD has the necessary resources, such as extra pads, cloths, etc., available in school. They employed tailored approaches based on the IQ level of the girls, such as demonstration, peer learning, visual aids, etc., to train AGDD to manage their menstruation independently.

### Theme 4: long term concerns of informal caregivers and perspectives on menstrual management

Informal caregivers are not only worried about the present challenges in managing menstruation, but they are equally worried about the future of their daughter. The uncertainty about who would take care of their child in their absence was a key worry expressed.

*“until and unless I am here I will look after her right? But what after me?”* (Participant 10, Informal Caregiver).

When asked about opinions on surgical options to stop menstruation, one informal caregiver reported that due to the fear of any sexual abuse in the future, she has consulted different doctors to know if there is an option to remove her daughter’s uterus.

*“I have almost consulted 10–15 doctors about her womb (uterus) … Can it be removed? Or are there any medicines. because if by mistakenly if at some point of time anyone does something to her….”* (Participant 4, Informal Caregiver).

However, the majority of informal caregivers opposed surgical options such as hysterectomy for their child. Their primary concerns were about the girl’s overall well-being, preserving their femininity, and future marriage prospects.

*“They’re not capable enough to take care of themselves. Just for three days of her periods. I don’t want to trouble her for a lifetime.”* (Participant 7, Informal Caregiver).

*“That is nonsense for her health. We will marry her to someone inshaahalaah* (if God wills) *everything good will happen with her.” (*Participant 6, Informal Caregiver).

Additionally, formal caregivers reported never advising informal caregivers to consider hysterectomy as a solution.

*“I have never advised anyone that you remove it(uterus) or you don’t remove it. There are advantages and disadvantages of getting it removed.”* (Participant 1, formal caregiver).

This theme emphasizes that while experiencing the present challenges, informal caregivers are equally worried about the future of their child, especially in their absence. Discussions about menstrual management revealed different views on permanent solutions such as hysterectomy. One informal caregiver cited that she has approached doctors to enquire if there is any possibility to remove her womb, as the caregiver was worried about any sexual abuse of her child in the future. However, the majority of the informal caregivers opposed the procedure, explaining concerns about the well-being of their child, femininity, and future marriage prospects. Formal caregivers had a neutral opinion on hysterectomy, without recommending it as a solution to any of the informal caregivers.

### Theme 5: recommendations given by informal and formal caregivers on supporting AGDD

Both informal and formal caregivers provided valuable recommendations based on their firsthand experiences, highlighting practical strategies and support systems that could improve the skills of the AGDD in managing their menstruation and improve the caregiving process, and address the challenges both the girls and the caregivers face. The recommendations suggested by the informal and formal caregivers are grouped under relevant subheadings, such as educating and training AGDD, communication and interaction among informal and formal caregivers, provision of quality menstrual products, infrastructure and institutional support, policy and financial protection.

### Educating and training AGDD

Informal caregivers emphasized the importance of teaching the AGDD about menstruation hygiene practices by a qualified expert.

“*There should be someone who gives these girls proper training. No one else does anything for them other than their parents.”* (Participant 9, Informal Caregiver)

Formal caregivers highlighted the need for premenstrual counselling when the girls are young, as girls are better prepared and have fewer challenges when they are informed beforehand about menstrual hygiene practices.

*“If we teach them in prior what to do during menstruation*,* how to wear a pad…. it would be easier later.”* (Participant 12, Formal caregiver)

### Communication and interaction among informal and formal caregivers

Formal caregivers shared that there should be clear communication and interaction between the formal and informal caregivers. They suggested that informal and formal caregivers should exchange their approaches to training the girls in menstruation management, allowing formal caregivers to better understand how to care for each girl and provide informal caregivers with valuable insights into the strategies used by formal caregivers, thereby improving the overall care and support of the girls.

*“Parents should meet caretakers*,* teachers and the way these girls are been given the training during their periods*,* this information must be told by the parents to the caretakers also.”* (Participant 8, Formal caregiver)

### Provision of quality menstrual products

Informal caregivers mentioned the need for provision of free high-quality sanitary pads and they shared that high quality pads reduce the stomach pain of the girls during menstruation.

“…*they should get good pads of good quality because of which the girl’s stomach pain should (stop)… because mainly stomach ache happens due to infection.* (Participant 13, Informal Caregiver)

### Infrastructure and institutional support

One informal caregiver emphasized the need for the Government to provide special infrastructure, such as hostels, for the AGDD.

*“They should do something for these kids*,* a hostel or something like that. They should organize everything for them in that hostel. That is my expectation from the government”* (Participant 3, Informal Caregiver)

### Policy and financial protection

Informal caregivers also pointed out the challenges in accessing appropriate health insurance coverage for children with special needs, noting that existing government schemes, such as Niramay Yojana [15], may not fully meet the specific healthcare requirements of these children.

*“There is nothing for these children*,* only one policy is there*,* only niramay yojana is there*,* that too you can only get 1 lakh* (100,000 Indian Rupees)*”.* (Participant 10, Informal Caregiver)

This theme highlights the different practical approaches to enhance the menstruation management skills of AGDD, as well as the necessary supports needed to improve the overall health and well-being of AGDD, as recommended by both informal and formal caregivers. Key recommendations included the need for expert-led menstrual hygiene education, pre-menstrual counselling, improved communication between formal and informal caregivers, provision of free high-quality menstrual products, and the need for infrastructure such as hostels and inclusive health insurance schemes for AGDD.

## Discussion

### Summary of findings

This study explored the perspectives of informal and formal caregivers of AGDD in menstruation management. An in-depth interview, using an IDI guide, was carried out, and using a combination of deductive and inductive thematic analysis, five themes, four deductive and one inductive, were identified. Deductive themes included: (1) challenges faced by informal caregivers in managing menstruation of AGDD, (2) patterns of menstrual acceptance and behavioural changes among AGDD, (3) long-term concerns of informal caregivers and perspectives of menstrual management, and (4) recommendations given by informal and formal caregivers on supporting AGDD. One inductive theme emerged from the data: (5) the role of formal caregivers in training AGDD in menstruation management.

### Implications of the findings in the context of existing research

The findings of this study highlight the experiences, opinions, challenges, and needs from the perspectives of both informal and formal caregivers in managing menstruation of AGDD. According to the study, AGDDs need specialized care and guidance to manage their menstruation and handle pubertal changes, aligning with previous studies conducted by Cummins et al. [8] in the United Kingdom (UK), Nurkhairulnisa AI et al. [11] in Malaysia, Karthikayini & Arun [9] in Pondicherry and Power et al. [16] in Bangladesh.

Informal caregivers often struggled with the girls’ inability to independently handle menstruation, leading to increased caregiving responsibilities, affecting the caregivers’ quality of life and freedom. Social embarrassment, especially in front of relatives and visitors, further complicated the caregiving experience. Prior studies by Wilbur J et al. [14] in Nepal and Dural O et al. [17] In Turkey, similar experiences have been reported by the caregivers of disabled girls.

Family support is crucial while caring for developmentally disabled girls. In a study by Thakur R & Mishra H [10] in Chandigarh, India, it was identified that mothers had the sole responsibility of caring for the disabled girl during menstruation and did not receive any support from their families. In the broader socio-cultural context of India, caregiving responsibilities, especially menstrual management, are traditionally considered as the tasks of women [18, 19]. While men’s involvement during menstruation is generally uncommon in India due to the cultural taboos and prevailing gender norms [20]. Our findings suggested that fathers were actively involved in the caregiving of AGDD during their menstruation. This shared responsibility reduced the burden on the mothers, highlighting the importance of family support in navigating the challenges of menstruation management for AGDD.

Unsurprisingly, the range of behavioural changes among AGDD during menstruation reported by informal caregivers (1 and 7) and formal caregiver 8 such as irritability, discomfort, and sometimes appeared settled are in line with the studies conducted among both the girls with developmental disabilities by Power R et al. [13] in Bangladesh, Wilbur J et al., [21] in Nepal, Flores-Medina M del R et al. [19] in Mexico and Retznik L et al. [22] in Germany as well the studies conducted among their typically developing peers by Ojezele M et al. [23] in Nigeria and Kumar A [24] in Tamil Nadu, India. The menstruation-related difficulties and difficulties experienced by AGDD are almost similar to those of their typically developing peers [25]. However, what differentiates AGDD from their typically developing peers is that their cognitive limitations may pose a barrier to learn the skills needed to manage menstruation [26]. Also, AGDD already face numerous difficulties due to their conditions, and menstruation further exacerbates this, demanding more support, adding to the burden on caregivers [9].

Unlike the findings from a study by Streur CS et al. [27] which found that the support and management of menstruation of disabled girls from school is challenging, the current study revealed that formal caregivers made greater efforts in training the girls to manage their menstruation independently. They used tailored teaching methods (such as verbally instructing, demonstrating, and peer learning) based on the intellectual impairment level of the girls, which not only facilitated menstrual hygiene and management but also promoted independence, reinforcing the importance of a structured and supportive environment in empowering AGDD in the special school setting.

While they are navigating through demanding caregiving responsibilities, informal caregivers are equally concerned about the future of their daughter. One informal caregiver added that she has approached doctors to know if there is any possibility to remove her daughter’s uterus as the caregiver was worried about any future sexual abuse. However, in the present study, the majority of the caregivers opposed permanent solutions such as the removal of the uterus due to various reasons, including concern about risks, well-being, femininity, and future marital prospects. These findings are consistent with earlier studies done by Power R et al. [16] in Bangladesh and Steward R et al. [28] in UK. This highlights the need for providing necessary counselling and support to informal caregivers to overcome the concerns and to make informed decisions about their daughters’ reproductive health. When considering counselling or educating informal caregivers about reproductive health, it is crucial to address any misconceptions they have about menstruation and menstrual products. As seen from the thoughts of participant 13, the belief that good-quality sanitary pads help to prevent infection and thereby reduce the menstrual pain reflects a misunderstanding that menstrual cramps are caused by infection, which is actually caused by hormonal variations [29]. Such misconceptions may influence how they seek solutions to manage the pain. This highlights a gap in reproductive health awareness, and addressing such misconceptions through tailored interventions helps caregivers to make more informed decisions in providing menstrual care to AGDD.

### Implications for policy and practice

The findings of the study suggest the need for tailored approaches for AGDD and their caregivers in managing menstruation and reducing the burden on caregivers. Firstly, it is crucial to provide psychological support services to informal caregivers to reduce their emotional stress associated with caregiving. Secondly, proper awareness should be given to informal caregivers on how to train AGDD to manage menstruation independently, thereby reducing the burden on caregivers. Reproductive health education is also crucial to eliminate misconceptions among caregivers. There is also a need to train AGDD with tailored approaches such as peer-learning, where the instructions are given by the caregiver to a menstruating girl, and the girl further shows the processes to her friends, as these girls learn quickly through imitations. Similarly, encouraging communication and interaction among informal and formal caregivers and exchanging the methods each of them uses to take care of AGDD can further help to enhance the care provided to AGDD. Lastly, policies should focus on improving access to health insurance and strengthening infrastructure to ensure that health services are inclusive, affordable, and accessible for individuals with developmental disabilities.

### Limitations

This study has a few limitations that should be acknowledged while interpreting the findings. First, the sample was drawn from a limited number of special schools within a specific geographic area, limiting the diversity in the study sample. Secondly, the one informal caregiver, who was not willing to participate, was caring for a girl with a very severe disability and expressed feeling overwhelmed due to the demanding caregiving responsibilities. This suggests that those experiencing intense caregiving burden may not be represented in our study sample and that our findings may not fully capture the experiences of caregivers facing the most extreme challenges. Lastly, although menstrual pain is commonly reported when studying menstruation management, it did not emerge during our study. This may be due to the absence of specific questions on pain in our interview guide. Future research should explore this by incorporating questions to understand how menstrual pain is expressed and managed among girls with developmental disabilities.

## Conclusion

This study highlights that informal caregivers faced various challenges due to the AGDD’s limited understanding of menstruation, inability to manage menstruation, behaviour changes associated with menstruation, and the need for constant supervision, which underscores the need for sustained support. Formal caregivers employed different strategies, such as demonstration-based training and peer learning in the school, to train the girls to manage their menstruation independently. Findings also point out deep concerns about the future of their child and diverse perspectives on surgical options such as hysterectomy. Overall, the study highlights a critical need for training AGDD, educating informal caregivers, fostering interaction between informal and formal caregivers, infrastructure support, and inclusive health policies, so that AGDD can be empowered and the burden associated with demanding caregiving responsibilities can be minimized.

## Supplementary Information

Supplementary Material 1.

## Data Availability

Access to the data of this study is restricted due to ethical and privacy concerns. Data will be available from the corresponding author upon reasonable request.

## Abbreviations

AGDD: Adolescent Girls with Developmental Disabilities
