# Supplementary material for: A qualitative study on the experiences of caregivers on menstrual management of adolescent girls with developmental disabilities in selected special schools of Karveer Taluka, Maharashtra
Source: BMC Womens Health. 2025 Oct 6;25:471. doi: 10.1186/s12905-025-04029-y (PMC12502475; doi:10.1186/s12905-025-04029-y)
Supplement: Supplementary file 1 — Supplementary Material 1. [file 12905_2025_4029_MOESM1_ESM.docx]

**Interview guide for informal caregivers**

***Opening Questions***

Good morning mam/sir. How are you?

I am Dr. Madhura Bhosale. I would like to discuss the experiences you encounter when your daughter/relative gets her periods. How do you manage? How does she manage? How are you balancing it with your regular life? I want to discuss your overall experience when it comes to her periods. Also, this interview will help other parents take care of the menstrual health of their developmentally disabled girl.

Can you please tell me about your daughter/relative? How old is she? Does she have any siblings? How old are they? How is she with her siblings? What are her hobbies? In which standard is she studying? What is her favorite subject? How is her bonding with you?

***Key Questions***

1. **Background and context**

Tell me about yourself. Do you live in a joint family or a nuclear family? How many family members are there in your family? What is your profession? What are your daily responsibilities? Can you give me a general idea about it?

1. **Child characteristics**
2. What were her reactions after the onset of her menarche? (happy, sad, stressed)
3. Did you notice any specific patterns in her behavior that you can attribute to her periods? If yes, what were they? Probe - irritation, depression, grief, anger
4. Did you notice any specific change in behavior when she attained adolescence? If yes, what were they? (Probe - shy, calm, restless, attraction towards opposite gender )
5. How is her behavior when you deliver menstrual care to her? e.g., during changing sanitary napkins? (Probe - acceptance or non-acceptance of the menstrual product, intolerant behavior.)
6. In your absence, does she let other family members deliver menstrual care for her? How is her behavior when they provide menstrual care for her? Is it the same as it is with you? (probe - while changing her sanitary napkin)
7. **caregiver strain/stress and intrapsychic factors**
8. When was her menarche? Where did it happen? Probe- school, home
9. What were your thoughts about her menstruation before she attained her menarche?

Probe - concern, fear, anxiety, happiness, stress.

1. What was your reaction after the onset of her menarche? How did you feel? (happy, sad, stressed)
2. How did your other family members react after they heard that she attained her menarche? (positive reaction, adverse reaction)
3. How did other societal members or relatives react after knowing about her menstruation? (probe -stigma, discrimination). Do you prefer talking about it with other society members in your life?
4. Did you encounter any challenges while delivering menstrual care to your daughter? What kind of challenges do you experience?
5. Can you describe a specific instance or incident where you were concerned or worried about her periods, and you remember it very well, even today?
6. **Coping factors**
7. Do you know of any surgery to stop her menstrual cycle permanently? Can you tell me about it?
8. Do you want to consider this option to stop her menstrual cycle? If yes, Why? If not, why?
9. What are the support measures you receive during her periods?

(Probe - doctor, psychiatrist, gynecologist, psychological or physical support, support from family members, relatives, information, advice)

1. How are school staff/teachers supporting her during her periods?
2. Have you made any plans for her future that will ensure her sustained menstrual health? What are they? (probe - nutritious food, health insurance plan)

***Closing Questions***

Do you want to recommend any other support measure for your daughter or for the informal caregivers that can help improve her menstrual health? (probe - government programs, free pads for caregivers, training programs for informal caregivers)

**Interview guide for formal caregivers.**

***Opening Questions***

Good morning mam/sir. How are you?

I am Dr. Madhura Bhosale. I would like to discuss the experiences/perceptions when she gets her periods. How do you manage? How does she manage? I want to discuss your overall experience regarding her period.

***Key Questions***

1. **Background and context**
2. Tell me about yourself. Do you live in a joint family or a nuclear family?
3. How many family members are there in your family?
4. What are your daily responsibilities? Can you give me a general idea about it
5. For how many years have you been working here as a teacher or school staff? How is your experience working here?
6. Tell me something about school.
7. Tell me about your students. Are these girls into any extracurricular activities? How is your relationship with your students?
8. Which training have you done for this profession?
9. **Child characteristics**
10. How is the behavior of menstruating girls in the class when they are on their periods? What are they?

Probe - irritation, depression, grief, anger

1. Are there any specific behavior changes you can attribute to their periods? What are they?
2. How do girls cooperate with the caretakers when they deliver menstrual care for them, e.g., during changing sanitary napkins?

Probe - acceptance or non-acceptance of the menstrual product, intolerant behavior.

1. **Coping factors**
2. Can you describe your role as a formal caregiver in supporting your students during menstruation?
3. What are the measures taken by schools to maintain the menstrual health of these girls? (probe- provision of pads,etc)
4. How do you make the girls understand the regular menstrual hygiene routines or self-help skills they should follow?
5. Do you face any challenges while training your students on menstrual hygiene management?
6. What methods do you use to teach girls about menstrual hygiene management? (probe- -demonstration, etc)? Can you share any such classroom activity where you taught girls how to manage their menstruation?
7. Do you conduct parent-teacher meetings regarding the menstruation of these girls? Can you tell me more about it? Why do you feel it is necessary to conduct such parent-teacher meetings?

***Closing questions***

Do you want to recommend any other support measure for your students or for the formal caregivers that can help improve her menstrual health? (probe - government programs, free pads for caregivers, training programs for informal caregivers)
